# Overexpressed Neuropilin-1 in Endothelial Cells Promotes Endothelial Permeability through Interaction with ANGPTL4 and VEGF in Kawasaki Disease

**DOI:** 10.1155/2021/9914071

**Published:** 2021-08-13

**Authors:** Junhua Huang, Shuwan Zhang

**Affiliations:** ^1^Medical Technology College, Xi'an Medical University, Xi'an, Shaanxi Province 710021, China; ^2^Department of Clinical Laboratory, The Affiliated Children's Hospital of Xi'an Jiaotong University, Xi'an, Shaanxi Province 710003, China

## Abstract

Disrupted endothelial permeability plays a crucial role in the vasculitis pathogenesis of Kawasaki disease (KD), which leads to pathological vascular leak and facilitates inflammatory cell infiltration in vascular lesions; however, the mechanisms involved in the development of endothelial barrier dysfunction during KD vasculitis are still largely unclear. Here, we found that sera from patients with KD can induce endothelial cell (EC) hyperpermeability compared to sera from healthy controls. We observed that serum vascular endothelial growth factor (VEGF) levels were increased in KD patients and sera from KD patients upregulated the expression of VEGF receptor 2 (VEGFR2) and neuropilin-1 (NRP1) in human coronary artery endothelial cells (HCAECs). Intriguingly, compared with silence of VEGFR2 in HCAECs, NRP1 silence resulted in a marked decrease in EC permeability. Furthermore, soluble NRP1 (sNRP1) remarkably reduced the stimulation of EC permeability by sera from KD patients compared with bevacizumab treatment. Importantly, we showed that besides VEGF, angiopoietin-like-4 (ANGPTL4), a NRP1-binding vasoactive factor, was also increased in KD and contributed to the EC permeability in KD conditions. In addition, levels of both ANGPTL4 and VEGF were inversely correlated with albumin levels in the serum of KD patients. Collectively, the data demonstrated that overexpressed NRP1, along with upregulated VEGFR2, in HCAECs treated with KD sera promotes endothelial permeability via interaction with the increased ANGPTL4 and VEGF in KD. Neutralization of hyperpermeability factors by sNRP1 may be a novel therapeutic strategy for KD vasculitis.

## 1. Introduction

Kawasaki disease (KD) is a common febrile pediatric disorder of unknown etiology and unclear pathogenesis with an increasing incidence and a leading cause of acquired heart disease in children under 5 years old [1]. KD is characterized by systemic medium-sized vascular inflammation, and coronary artery lesions (CALs) are the main complication of KD vasculitis. If there is no timely high-dose intravenous immunoglobulin (IVIG) therapy, CALs could occur in ~30% KD children and bring higher hospitalization costs [2]. Although the exact molecular mechanism of KD vasculitis still remains obscure, clinical and experimental evidence has shown that KD vasculitis is closely associated with increased vascular permeability that leads to vascular leakiness, decreased plasma proteins, and inflammatory cell infiltration of endothelium [3]. In the development of vascular leakage, vascular endothelial cell (EC) hyperpermeability induced by vasoactive factors has been shown to play a pivotal role [4]. Among the vasoactive factors, vascular endothelial growth factor (VEGF) is the most well-described pathological hyperpermeability mediator; by binding with its receptors on ECs, VEGF exerts a powerful promotion of vascular permeability, which may contribute to the leakage of substances in plasma, such as albumin, and the subendothelium infiltration of blood cells in KD [5]. In fact, several studies have shown that VEGF and its receptors, including VEGF receptor 1 (VEGFR1), VEGFR2, and neuropilin-1 (NRP1), are upregulated in acute KD blood vessels and VEGF levels are reversely correlated with albumin concentrations in KD condition [6, 7]. Despite the evidence supporting a correlation of VEGF with vascular leakage in KD, the molecular signaling events that explain its mechanism of action in the induction of vascular hyperpermeability are largely unknown and whether other vasoactive mediators also participate in the EC permeability in KD still remains unclear.

NRP1, a transmembrane glycoprotein, acts as a coreceptor for VEGF, which enhances VEGF-VEGFR2-mediated bioactivity, such as angiogenesis and endothelial permeability [8]. On the other hand, besides binding VEGF, as an essential cell-surface protein, NRP1 has also been shown to interact with semaphorins and play pivotal functions in different cells. For instance, semaphorin3A-NRP1 interaction is involved in not only the regulation of axon repulsion in neurocyte but also the promotion of disruption of endothelial integrity in ECs [9]. Interestingly, more recently, a study reported that angiopoietin-like-4 (ANGPTL4), a secreted adipokine with an important role in lipid metabolism, can promote endothelial permeability by directly interacting with NRP1 on ECs [10]. Based on the above evidence, we hypothesized that in addition to VEGF, other NRP1-binding vascular permeability factors may also contribute to the development of disrupted endothelial permeability in KD.

In the present study, we found that sera from patients with KD can induce human coronary artery endothelial cells (HCAECs) to upregulate the expression of VEGFR2 and NRP1. The overexpression of these two receptors, along with the increased ANGPTL4 and VEGF in the serum of KD, is responsible for the EC hyperpermeability. Moreover, compared with bevacizumab (a VEGF-neutralizing antibody), soluble NRP1 (sNRP1), via neutralization of both ANGPTL4 and VEGF in KD sera, remarkably reduced the stimulating effect of KD sera on EC permeability. Our study indicated that ANGPTL4 is implicated in the promotion of vascular permeability in KD, and sNRP1 may be a novel therapeutic candidate for the treatment of KD vasculitis.

## 2. Subjects and Methods

### 2.1. Human Subjects

This study was approved by the Ethics Committee of the Affiliated Children's Hospital of Xi'an Jiaotong University and was conducted in accordance with the 2013 Declaration of Helsinki. Written informed consent was obtained from the parents of all participants included in this study. All patients diagnosed with KD met the criteria of “Diagnosis, Treatment, and Long-Term Management of Kawasaki Disease” by the American Heart Association in 2017 [11]. In this study, serum samples of 25 patients with acute KD before IVIG treatment (none of them had CALs) and 15 healthy children were collected from the Affiliated Children's Hospital of Xi'an Jiaotong University (Xi'an, China) between June 2020 and December 2020. To minimize the impact of platelet activation on the serum components during blood collection, whole blood samples were taken into sterile silicized glass tubes before IVIG and aspirin treatment from the antecubital vein by an experienced nurse with 20-gauge needles in 1 minute and without using a tourniquet or flapping the blood collection site. Serum samples were obtained from whole blood by centrifuging for 10 min at 300 × *g* and then filtered by sterilizing filters and stored at -80°C until use.

### 2.2. Serum Sample Detection

The levels of VEGF, ANGPTL4, and semaphorin3A in KD serum were measured by 2-step sandwich ELISA kits (ELK Biotechnology, Wuhan, China) according to the manufacturer's instructions. Serum albumin concentration was assessed by the automatic biochemical analyzer (Beckman Coulter, USA). Serum samples were analyzed in duplicate.

### 2.3. Cell Culture and Reagents

Human coronary artery endothelial cells (HCAECs) were purchased from SCIENCELL (CA, USA) and cultured as previously described [12]. For the human serum stimulation assay, HCAECs were cultured in RPMI-1640 media containing 20% KD serum or HC serum for 6 hours. Recombinant human ANGPTL4 (rANGPTL4), bevacizumab biosimilar, and sNRP1 (recombinant human NRP1-Fc protein) were purchased from R&D Systems. IVIG was purchased from Rongsheng Pharmaceutical Co. Ltd. (Chengdu, China). siRNA of control, VEGFR2, and NRP1 were designed and synthesized by Sangon (Shanghai, China). siRNA transfection into HCAECs was performed using Lipofectamine 3000 reagent (Invitrogen, USA).

### 2.4. Quantitative Real-Time PCR (qRT-PCR)

Total RNA was extracted from HCAEC lysates by RNeasy mini kit (Qiagen, Germany). cDNA was synthesized with PrimeScript RT Mix (Takara, Japan). The mRNA expression levels of VEGFR1, VEGFR2, NRP1, and NRP2 were analyzed using SYBR Green Real-Time PCR Mix (Takara, Japan) in an ABI 7500 analyzer (ABI, USA).

### 2.5. EC Permeability Assays

HCAECs were seeded on transwells (6.5 mm diameter polycarbonate membranes with 0.4 *μ*m pore, Corning, USA) that were coated with fibronectin for 1 h and allowed to grow as 2-day-old monolayers. After serum-starvation overnight, 500 *μ*l and 200 *μ*l media containing 20% human serum (KD serum or HC serum) were added for 6 h to the lower and upper chambers, respectively. After rinsing, a total of 200 *μ*l 300 *μ*g/ml FITC-dextran (Mw = 10000, Sigma) was added to the upper chamber for 30 min. The fluorescence intensity in the lower chamber was measured using a fluorescence microplate reader (PerkinElmer, USA).

### 2.6. Statistical Analysis

Quantitative data were expressed as the means ± standard deviation (*M* ± SD). Unpaired Student's *t-*test was used to determine statistical differences between two groups. Pearson's method was performed to explore the correlation between levels of VEGF and ANGPTL4 and the concentration of albumin in KD serum. A 2-tailed *P* value < 0.05 was statistically significant. Statistical analysis was conducted using Prism 7.0 software (GraphPad, USA).

## 3. Results

### 3.1. Vascular Permeability Is Higher in the KD Group

**Figure 1:**
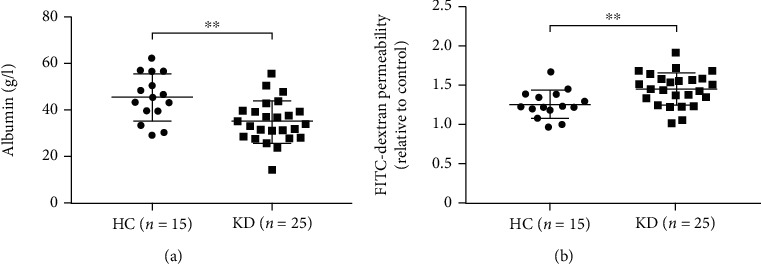
Vascular permeability is higher in the KD group. (a) Levels of serum albumin in healthy control (HC) children and acute KD (KD) patients. (b) Induction of permeability of HCAEC monolayers by medium containing 20% serum from HC children or KD patients. HCAEC monolayers treated by medium containing 10% fetal bovine serum (FBS) as control. ^∗∗^*P* < 0.01.

Hypoalbuminemia, a condition caused by albumin leakage outside the blood vessels following high vascular permeability, is a characteristic feature of KD [13]. So we first compared the concentrations of albumin between KD patients and HC individuals. Consistent with previous studies [5, 7], albumin level was significantly lower in the KD group than in the HC group (Table 1 and Figure 1(a)), suggesting a vascular hyperpermeability situation presented in KD patients. Furthermore, to quantitate the promotion of vascular permeability in KD, we obtained serum from patients with KD and performed EC permeability assays using HCAEC monolayers. We observed a marked increase in the promotion of EC permeability upon exposure of HCAEC monolayer to serum from KD patients compared with HC children (Figure 1(b)).

### 3.2. VEGF Is Increased in KD Serum and Levels of VEGFR2 and NRP1 Expression Are Elevated in ECs Treated with Sera from KD Patients

**Figure 2:**
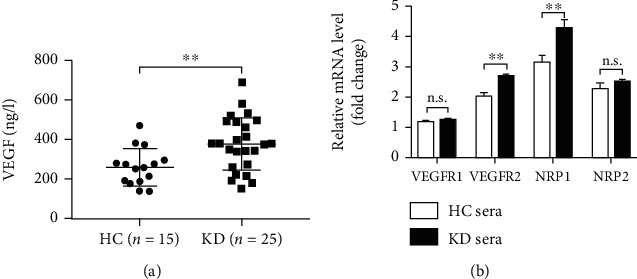
VEGF is increased in the KD group, and KD sera promote VEGFR2 and NRP1 expression of HCAECs. (a) Levels of VEGF in the HC group and the KD group. (b) Effect of human sera on mRNA levels of VEGFRs and NRPs in HCAECs. HCAECs were treated by medium containing 20% sera from healthy children (HC sera; pooled from 10 individuals) or from KD patients (KD sera; pooled from 10 patients). The mRNA level of each gene was normalized to that of *β*-actin. Data are expressed as *M* ± SD from 3 separate experiments. ^∗∗^*P* < 0.01; n.s.: not significant.

To explore the mechanism by which KD serum induces EC hyperpermeability, we next measured the levels of VEGF, a well-known vascular permeability factor, in KD patients and the expression of four VEGF receptors, VEGFR1, VEGFR2, NRP1, and NRP2, in HCAECs stimulated by KD sera. We found that serum VEGF levels were higher in KD patients than in HC children (Table 1 and Figure 2(a)). We also observed a market increase in the mRNA levels of VEGFR2 and NRP1 in HCAECs treated with KD sera compared with HCAECs treated with HC sera; on the other hand, despite a slight elevation in the mRNA levels of VEGFR1 and NRP2 in KD sera-treated HCAECs, there was no statistical significance in the expression of the two receptor genes between the KD group and the HC group (Figure 2(b)), indicating that VEGFR2 and NRP1, but not VEGFR1 and NRP2, contributed to the promotion of EC permeability in KD conditions.

### 3.3. Downregulation of VEGFR2 or NRP1 Attenuates the EC Permeability

**Figure 3:**
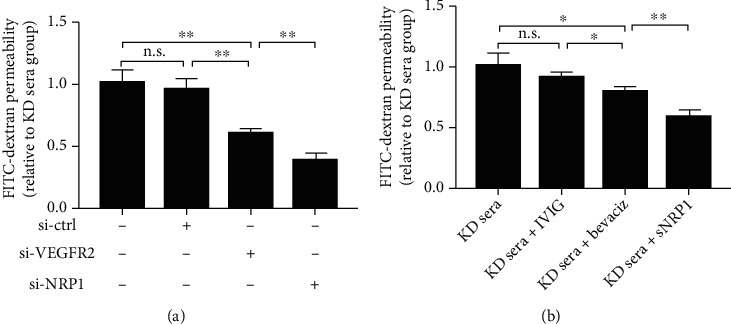
Downregulation of VEGFR2 or NRP1 blocks KD sera-induced hyperpermeability of HCAEC monolayers. (a) EC permeability assay upon transfection of control siRNA, VEGFR2 siRNA, or NRP1 siRNA and stimulation with medium containing 20% KD sera (pooled from 10 KD patients). (b) Effect of KD sera incubated with the indicated pharmacologic agent on EC permeability. Data are expressed as *M* ± SD from 3 separate experiments. ^∗^*P* < 0.05; ^∗∗^*P* < 0.01; n.s.: not significant.

We next set out to investigate whether the upregulation of VEGFR2 and NRP1 is responsible for the KD sera-induced EC hyperpermeability. To this end, we blocked the expression of VEGFR2 and NRP1 with specific siRNAs in HCAECs. We found that upon the knockdown of VEGFR2 or NRP1 expression on HCAECs, the enhancement of EC permeability induced by KD sera was significantly reduced (Figure 3(a)). However, interestingly, compared with silence of VEGFR2, NRP1 silence in HCAECs resulted in a more marked decrease in EC permeability, which prompted us to hypothesize that other permeability factors, besides VEGF, in KD sera may be involved in the promotion of EC permeability by directly binding NRP1 on HCAECs. To further verify our hypothesis, we incubated KD sera using bevacizumab and sNRP1, respectively, and then treated HCAEC monolayers with these KD sera. As expected, sNRP1 remarkably reduced the promotion effect of KD sera on EC permeability compared with bevacizumab (Figure 3(b)).

### 3.4. ANGPTL4 in KD Sera Contributes to the Hyperpermeability of Endothelial Monolayer by Binding to NRP1

**Figure 4:**
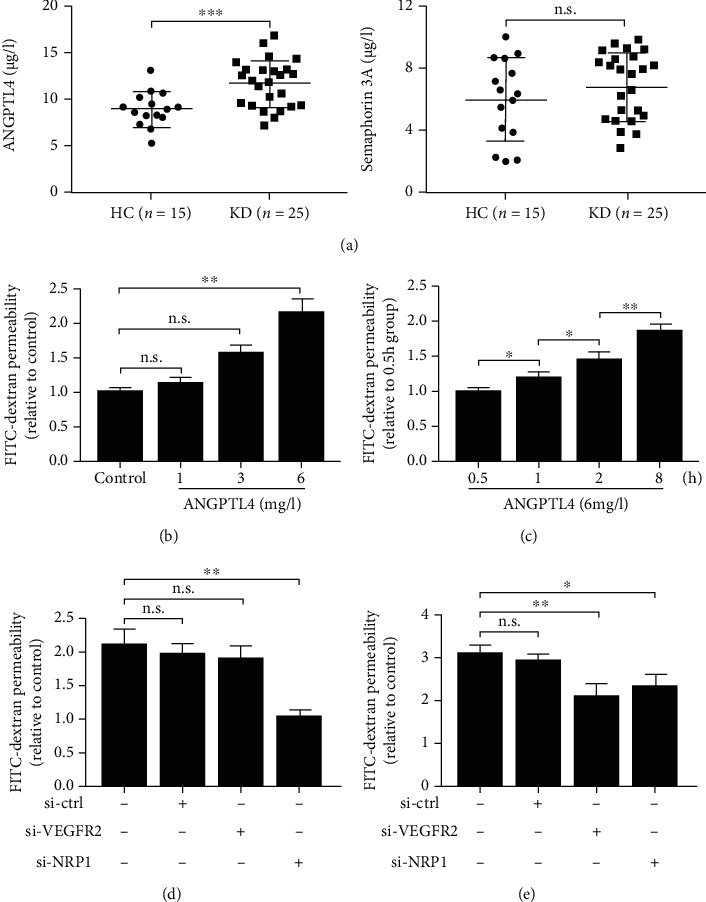
ANGPTL4 contributes to the permeability of HCAEC monolayers by binding NRP1. (a) Levels of ANGPTL4 and semaphorin3A in the HC group and the KD group. (b, c) EC permeability assay upon treatment of HCAECs with different doses of rANGPTL4 or with 6 mg/l rANGPTL4 in different times. EC permeability assay upon transfection of control siRNA, VEGFR2 siRNA, or NRP1 siRNA and stimulation with (d) 6 mg/l rANGPTL4 for 8 h or (e) 60 *μ*g/l VEGF for 6 h. PBS treatment as control. Data are expressed as *M* ± SD from 3 separate experiments (b–e). ^∗^*P* < 0.05; ^∗∗^*P* < 0.01; ^∗∗∗^*P* < 0.001; n.s.: not significant.

ANGPTL4 and semaphorin3A, two NRP1-binding proteins, have been reported to act as vasoactive mediators for endothelium permeability [9, 10, 14]. We therefore measured the levels of the two proteins in KD and found that the levels of ANGPTL4, but not semaphorin3A, were especially elevated in KD patients compared with HC (Table 1 and Figure 4(a)). Furthermore, to determine whether the ANGPTL4/NRP1 axis is implicated in the promotion of EC permeability, we treated HCAEC monolayer with recombinant ANGPTL4 (rANGPTL4). As shown in Figures 4(b) and 4(c), rANGPTL4 enhanced endothelial permeability in a dose- and time-dependent manner. Moreover, silencing of NRP1, but not VEGFR2, significantly inhibited the rANGPTL4-induced hyperpermeability of HCAEC monolayer (Figure 4(d)). Conversely, the promotion of HCAEC permeability by VEGF that bind both VEGFR2 and NRP1 was attenuated by the knockdown of either VEGFR2 or NRP1 (Figure 4(e)).

### 3.5. ANGPTL4 and VEGF Are Correlated with Low Albumin Level in KD

**Figure 5:**
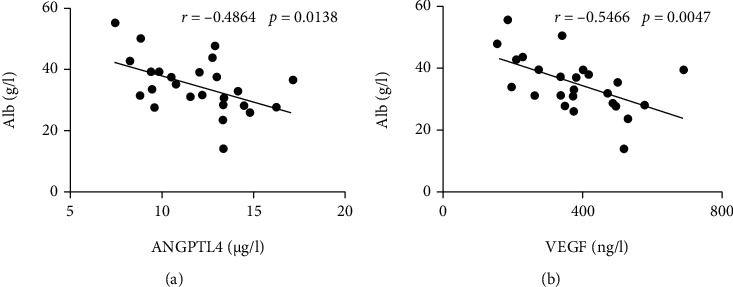
The relationship between ANGPTL4 or VEGF and albumin in KD. (a) Correlation of levels between ANGPTL4 and albumin in KD patients. (b) Correlation of levels between VEGF and albumin in KD patients. Pearson's test was performed to explore the correlation.

To further identify if both ANGPTL4 and VEGF contribute to the hyperpermeability conditions of KD, we analyzed the relationship of the concentrations between the two proteins and albumin in KD sera and found that the levels of both ANGPTL4 and VEGF negatively correlated with albumin levels in the serum of KD patients, which further indicated that the two vasoactive factors are responsible for vascular permeability in KD conditions (Figure 5).

## 4. Discussion

Increased vascular permeability is a significant feature of KD pathophysiology, but its underlying mechanism is still largely unclear. Here, we demonstrated that overexpressed NRP1 and VEGFR2 in ECs contribute to the vascular hyperpermeability by binding with ANGPTL4 and VEGF in KD and highlighted the pathological involvement of ANGPTL4/NRP1 interaction in the promotion of EC permeability in KD conditions. Furthermore, our observations showed a potential therapeutic effect of sNRP1 on KD vasculitis.

Hypoalbuminemia is common and thought to be an indicator of the increased vascular permeability in KD [7, 15]. In this study, we also found that albumin concentrations were decreased in the KD group, indicating these KD patients presented a status of vascular leakage. Sera from patients with KD have recently been used as a stimulator to achieve KD vasculitis *in vitro* [12, 16–18]. KD serum can induce dysfunction and inflammation of HCAECs by activating Ca2+/nuclear factor of activated T cell signaling pathway, which leads to the proliferation and angiogenesis of endothelial cells [16]; and in our previous study, we showed that sera from KD patients can stimulate HCAECs to upregulate some cell-membrane molecules, such as plexin Bs [12]. Here, for the first time, we treated HCAEC monolayers with KD sera and investigated the effect of KD sera on EC permeability. Thrillingly, we observed that EC monolayers exhibited an enhanced permeability upon KD sera stimulation compared with HC sera treatment, which prompted us to further explore the mechanism underlying this phenomenon.

VEGF, also known as VEGF-A, is a well-known vascular permeability factor and acts as a proinflammatory cytokine by increasing the permeability of ECs [4, 19]. Accumulating and compelling evidence has supported a role of VEGF in the pathogenesis of KD vasculitis [5–7, 20–22]. Herein, similarly, the levels of VEGF in KD sera were higher than those in HC sera, indicating this molecule may be responsible for the KD sera-induced hyperpermeability of the HCAEC monolayers. In KD, the source of increased VEGF in KD serum and whether the high product of VEGF is a primary or secondary event are largely unknown. One possibility is that inflammatory cells, such as macrophages, which are usually activated in KD conditions, overexpress and secrete VEGF. Another possibility is that VEGF sequestered in the extracellular matrix can be released to peripheral circulation upon the disruption of extracellular matrix and the action of increased proteases, such as matrix metalloproteinase 9, in KD situations [19]. VEGF plays its bioactivity mainly by interacting with its receptors, such as VEGFR and NRP, on the target cells [23]. So we comprehensively analyzed the expression of VEGF receptors, including VEGFR1, VEGFR2, NRP1, and NRP2, on HCAECs after KD sera treatment and found increased expression levels of VEGFR2 and NRP1 in KD sera-stimulated HCAECs. Of note, Yasukawa et al.'s study using autopsy samples has shown that the expression of NRP1 and VEGFG2 is increased in coronary vessels but the upregulation of the two receptors is only limited to the early stage of acute KD [6]; similarly, an animal model of KD-CALs also demonstrated an early overexpression of VEGF in blood vessels [24]. Indeed, all the serum samples collected to induce the permeability of HCAEC monolayers in this study were from acute KD patients in the early stage (fever less than 5 days). On the other hand, despite a slight elevation in the expression of VEGFR1 and NRP2, the changes were comparable with those of the HC group, indicating VEGFR1 and NRP2 were not the main receptors for VEGF-mediated bioactivities in the early stage of KD. Therefore, it can be speculated that VEGF-induced vascular hyperpermeability via interaction with VEGFR2 and NRP1 may be an early or even an initial event in the pathogenesis of KD vasculitis as a consequence of earlier immune-inflammatory change in the human body, but prior to the development of KD-CALs.

Intriguingly, the knockdown of NRP1 expression in HCAECs showed a marked decrease in HCAEC monolayer hyperpermeability induced by KD sera compared with VEGFR2 knockdown, suggesting an additional combination between NRP1 and its ligands that contribute to NRP1-mediated EC permeability. Besides binding VEGF, NRP1 has been shown to interact with two other vascular permeability factors, semaphorin3A and ANGPTL4 [9, 10, 14]. In the present study, we found that the levels of ANGPTL4 were significantly increased in the KD group, whereas semaphorin3A levels had no significant change between KD and HC, which indicated that ANGPTL4 may also participate in KD sera-stimulated EC hyperpermeability via interaction with NRP1.

Despite a well-described key effect of ANGPTL4 on lipid metabolism, a lot of research evidence has shown that ANGPTL4, as a multifunctional protein, plays an important role in vascular homeostasis and inflammation [25, 26]; however, the role of ANGPTL4 in the promotion of vessel permeability has been controversial. Some studies showed that ANGPTL4 could suppress vessel permeability and preserve endothelial integrity [27, 28], while some researches supported a role of ANGPTL4 in weakening EC-EC junctions, disrupting endothelial barrier, and increasing vascular leakage [10, 14, 22]. Interestingly, Sodhi et al.'s study on diabetic macular edema recently demonstrated that ANGPTL4/NRP interaction can independently induce the permeability of human umbilical vein endothelial cell (HUVEC) and retinal endothelial cell (REC) monolayers [10]. Although it is thought that the different effect of ANGPTL4 on vascular permeability may be cell-, tissue-, or context-specific and HUVECs and HCAECs show different biobehaviour in the same condition [29], consistent with Sodhi et al.'s study [10], we also found an enhanced permeability upon ANGPTL4 in HCAEC monolayers, indicating a broad vascular activity of ANGPTL4 on different endothelial cells. Furthermore, similar to the relationship between VEGF and albumin showed in this study and previous researches [5, 7], a negative correlation of ANGPTL4 and albumin was also found in our study (Figure 5(a)), further suggesting the implication of ANGPTL4 in vascular leakage of KD. Moreover, our results proved again that NRP1 is necessary for VEGF-mediated vessel permeability (Figure 4(e)), further suggesting that NRP1 interacts with both ANGPTL4 and VEGF, promotes vascular permeability, and in turn could contribute to the development of KD-CALs. However, the source of high ANGPTL4 in KD serum is unclear. It is known that in humans, ANGPTL4 is produced by adipose tissue, heart, and small intestine [25], and all these tissues have been reported to be dysfunctional in KD conditions [30–32]. So, in the future, it is worthy to explore the specific tissue source of ANGPTL4 in KD.

Of note, sNRP1 showed a marked inhibition effect on KD sera-induced EC hyperpermeability. Despite a similar inhibition by bevacizumab, this VEGF-neutralizing antibody was not as powerful as sNRP1, which therefore indicated sNRP1 may be a promising therapeutic candidate for KD treatment and prevent the inflammatory progress of vessels at an earlier stage of acute KD. Additionally, it is noteworthy that IVIG treatment showed no significant inhibition of KD sera-induced EC hyperpermeability, which is similar to Terai et al.'s study [7] showing that VEGF levels were still high after IVIG therapy, indicating that IVIG might have no effect on controlling vascular permeability.

There are some limitations in this study. First, we did not explore what factors in KD serum contribute to the overexpression of VEGF receptors in HCAEC. In this regard, some proteins, such as CRP and TNF-*α* [33, 34], have been shown to regulate the expression of VEGFRs and NRPs of ECs; however, whether other molecules also participate in the upregulation of these receptors in KD situations needs to be investigated in the future. Second, NRP1 has also been shown to bind with other factors, such as fibroblast growth factor, transforming growth factor, and platelet-derived growth factor [35–37]. Whether these NRP1-binding molecules are also involved in vascular permeability in KD conditions is unclear. Last, due to the usage of sera from acute KD patients at the early stage of disease and the heterogeneity of serum composition, the smaller sample size in the current study might weaken our conclusions and studies with a larger sample size and a broader serum source (from individuals at different stages of KD) are needed in the future.

## 5. Conclusions

In summary, our study showed that both the upregulation of NRP1 and VEGFR2 in ECs and the increase of ANGPTL4 and VEGF in serum are responsible for the vascular hyperpermeability in KD setting. For the first time, we found that the ANGPTL4/NRP1 axis participates in the promotion of EC permeability in KD conditions. sNRP1 has a potential as a novel therapeutic candidate for KD treatment by neutralizing ANGPTL4 and VEGF and inhibiting vascular hyperpermeability.

## Figures and Tables

**Table 1:** Comparison of baseline characteristics between KD patients and healthy controls.

Groups	Kawasaki disease	Healthy control	*P* value
Number	25	15	
Male	19	11	
Female	6	4	
Age (months)	30.52 ± 15.63	27.70 ± 22.42	0.6420
Albumin (g/l)	35.00 ± 8.90	45.43 ± 10.03	0.0015
VEGF (ng/l)	379.30 ± 133.90	259.50 ± 93.79	0.0043
ANGPTL4 (*μ*g/l)	11.87 ± 2.54	9.08 ± 1.91	0.0007
Semaphorin3A (*μ*g/l)	6.79 ± 2.21	6.02 ± 2.62	0.3228

Data are expressed as means ± standard deviation for normally distributed data or number for categorical variables.

## Data Availability

The data used to support the findings of this study are included within the present article. The raw data are available from the corresponding author upon request.
